# Development of a Multicomponent Intervention to Prevent Alzheimer's Disease

**DOI:** 10.3389/fneur.2019.00490

**Published:** 2019-05-08

**Authors:** Satoshi Saito, Yumi Yamamoto, Masafumi Ihara

**Affiliations:** ^1^Department of Neurology, National Cerebral and Cardiovascular Center, Suita, Japan; ^2^Research Fellow of Japan Society for the Promotion of Science, Tokyo, Japan; ^3^Department of Pediatric Dentistry, Osaka University Graduate School of Dentistry, Suita, Japan; ^4^Department of Regenerative Medicine and Tissue Engineering, National Cerebral and Cardiovascular Center, Suita, Japan

**Keywords:** Alzheimer's disease, cerebrovascular disease, cerebral amyloid angiopathy, MIND diet, glymphatic system, IPAD, cilostazol

## Abstract

Recent advances in vascular risk management have successfully reduced the prevalence of Alzheimer's Disease (AD) in several epidemiologic investigations. It is now widely accepted that cerebrovascular disease is both directly and indirectly involved in AD pathogenesis. Herein, we review the non-pharmacological and pharmacological therapeutic approaches for AD treatment. MIND [Mediterranean and DASH (Dietary Approaches to Stop Hypertension) Intervention for Neurodegenerative Delay] diet is an important dietary treatment for prevention of AD. Multi domain intervention including diet, exercise, cognitive training, and intensive risk managements also prevented cognitive decline in the Finnish Geriatric Intervention Study to Prevent Cognitive Impairment and Disability (FINGER) study. To confirm these favorable effects of life-style intervention, replica studies are being planned worldwide. Promotion of β-amyloid (Aβ) clearance has emerged as a promising pharmacological approach because insufficient removal of Aβ is more important than excessive Aβ production in the pathogenesis of the majority of AD patients. Most AD brains exhibit accompanying cerebral amyloid angiopathy, and Aβ distribution in cerebral amyloid angiopathy closely corresponds with the intramural periarterial drainage (IPAD) route, emphasizing the importance of Aβ clearance. In view of these facts, promotion of the major vascular-mediated Aβ elimination systems, including capillary transcytosis, the glymphatic system, and IPAD, have emerged as new treatment strategies in AD. In particular, the beneficial effects of cilostazol were shown in several clinical observation studies, and cilostazol facilitated IPAD in a rodent AD model. The COMCID (Cilostazol for prevention of Conversion from MCI to Dementia) trial, evaluating the efficacy of cilostazol for patients with mild cognitive impairment is currently ongoing in Japan. Such therapeutic approaches involving maintenance of cerebrovascular integrity and promotion of vascular-mediated Aβ clearance have the potential to be mainstream treatments for sporadic AD.

## Introduction: Alzheimer's Disease and Cerebrovascular Disease

The pathogenesis of cognitive impairment in Alzheimer's disease (AD) cannot be simply explained by the neurodegeneration induced by amyloid β (Aβ) and tau accumulation, although they are the most established characteristics of AD pathology (1, 2). Because these characteristics are detectable by pathological examination, and do not always correlate with cognitive function, antemortem clinical diagnosis of AD is often denied by postmortem diagnosis, while individuals clinically diagnosed as cognitively intact are occasionally found to have AD pathology at postmortem (3).

Accumulating evidence suggests that cognitive impairment in AD patients, especially in elderly subjects, is attributed to both neurodegeneration and cerebrovascular disease (CVD) (4–6). Mixed AD/CVD pathology is commonly seen in patients with clinically diagnosed AD (7, 8). CVD itself impairs cognitive function, increasing the risk of progressing to clinically defined dementia (9–11). More importantly, recent studies indicate that vascular lesions are directly involved in AD pathogenesis (12). One of the mechanisms linking CVD to AD is decreased cerebral blood flow (13), which modulates amyloid precursor protein (APP) cleavage enzymes, such as β- and γ-secretase, leading to increased Aβ production (14). Chronic cerebral hypoperfusion also accelerates tau pathologies (15). Further, in a transgenic AD mouse model, the majority of parenchymal Aβ plaques were deposited adjacent to cerebral blood vessels, implying the involvement of the cerebral vasculature in Aβ accumulation (16). In addition to animal models, a pivotal role of CVD in AD pathogenesis has also been revealed by clinical studies. Cerebrovascular injury, including blood brain barrier (BBB) dysfunction (17, 18) and decreased cerebral blood flow (19), was found to precede neurodegeneration in AD patients. As such, the importance of vascular contributions to AD is now emphasized in a scientific statement from the Alzheimer's Association (20), and the close interrelationship between neurodegeneration and CVD has become a major focus of recent research in dementia (21, 22).

## Lifestyle interventions to Prevent Cognitive Impairment

### Management of Vascular Risk Factors

AD and CVD share several risk factors (23, 24). For example, hypertension, diabetes mellitus, and dyslipidemia are related to the risk of progression to dementia (25, 26). Conversely, intensive management of vascular risk factors was associated with reduced risk of dementia (25), as well as amelioration of cognitive decline in AD patients (27). Several epidemiologic reports have also revealed a decrease in dementia prevalence over the last few decades, which is considered to be, at least in part, attributable to well-controlled vascular risk factors (28–32). Thus, the importance of risk controls in AD is established.

### The FINGER Study

Although pharmacological intervention is a fast and effective approach in treating vascular risk factors, lifestyle intervention remains important for prevention and treatment of AD and CVD. A population based randomized controlled trial, the Finnish Geriatric Intervention Study to Prevent Cognitive Impairment and Disability (FINGER) study clearly revealed the importance of life-style modification (33). In that study, a multi domain intervention successfully prevented cognitive decline in elderly subjects. The multi domain intervention consisted of nutritional guidance, exercise, cognitive training, and social activity, while the control group received regular health advice. Metabolic and vascular risk factors were carefully monitored in the intervention group, and when the adjustment of pharmacologic treatment was needed, participants were recommended to contact primary health care centers (34).

Interestingly, the beneficial effects in the FINGER study were not modified by baseline status of sociodemographic, socioeconomic status, cognitive function, cardiovascular factors, or cardiovascular comorbidity, indicating that life-style intervention is universally effective in the elderly (35). Of all the subdomains of cognitive function, substantial positive effects were found in executive function and processing speed (33). However, because no pathological analysis was performed in that study, it remains unclear whether lifestyle intervention has any effect on reducing Aβ and tau pathologies and reducing neurodegeneration. Ongoing replica studies around the world may answer this important question (36).

### MIND Diet

In the FINGER study, the intervention group was recommended to consume whole grains, fruits, vegetables, and oils, while low-fat options were recommended over milk and meat products (34). These foods are well-known components of the Mediterranean diet (37). Improvement in cognitive function with the Mediterranean diet was shown in a multicenter randomized clinical trial for community-dwelling people with vascular risk factors (38). The Dietary Approach to Systolic Hypertension (DASH) diet, which aims to reduce blood pressure, was also reported to prevent dysexecutive function in patients with hypertension and obesity (39). Combining these two diets, the Mediterranean and DASH Intervention for Neurodegenerative Delay (MIND) diet was proposed in the Rush Memory and Aging project in the United States. The MIND diet places a strong emphasis on natural, plant-based food, specifically promoting increased consumption of berries and green leafy vegetables, with limited intake of animal-based and highly saturated fat foods (40). Surprisingly, the incidence of AD in participants who strictly followed the MIND diet was reduced by half compared with those who did not follow it (41). The improvement in cognitive function was particularly significant in the domains of episodic memory, semantic memory, and perceptual speed (42). Replica studies are required to verify these favorable effects (43), although some modification may be required when the MIND diet is applied to other countries. For example, larger fish consumption and excessive salt intake are more common in East Asian than in Western countries. Thus, taking the local food culture into consideration is important when designing a feasible diet therapy (Table 1).

**Table 1:** Original and Japanese-modified version of the MIND diet.

	**Original version**	**Japanese version**
Green leafy vegetables	≥6 servings/week	≥6 servings/week
Other vegetables	≥1 serving/day	≥1 serving/day
Berries	≥2 servings/week	Strawberries, ≥2 servings/week
Nuts	≥5 servings/week	≥5 servings/week
Olive oil	Primary oil used	Primary oil used
Butter, Margarine	< 1 tablespoon/day	Minimum amount
Cheese	< 1 serving/week	< 1 serving/week
Whole grains	≥3 servings/day	Brown rice, ≥3 servings/day
Fish (not fried)	≥1 meal/week	Primary choice
Beans	>3 meals/week	>3 meals/week
Poultry (not fried)	≥2 servings/week	≥2 servings/week
Red meat products	< 4 servings/week	< 4 servings/week
Fast fried foods	< 1 time/week	< 1 time/week
Pastries and sweets	< 5 servings/week	< 5 servings/week
Wine	1 glass/day	Wine or green tea

*The Japanese version of the MIND diet was modified from the original edition (41). These changes were introduced because berries and whole grains are less available in Japan than in Western countries. In Japan, more strict salt restriction is needed; most Japanese eat fish more than once a week, and low alcohol tolerance is more common in Japanese*.

## Pharmacological Interventions for the Promotion of Aβ Clearance

### Vascular Mediated Aβ Elimination

The amyloid hypothesis suggests that Aβ precedes tau pathology (44). Both Aβ overproduction and elimination failure have been demonstrated as a cause of AD (45), although the latter plays a major role in the pathogenesis of sporadic AD, which is especially more common in the elderly (46, 47). Thus, promotion of Aβ clearance has been proposed as a new therapeutic approach for sporadic AD (48).

In mice, >80% of ^125^I-labeled Aβ_40_ was found in the blood or cerebrospinal fluid (CSF) at 5 h after Aβ_40_ injection into the caudate nucleus, with minimal remaining in the brain parenchyma (49). Several mechanisms of Aβ clearance have been identified (50), with the major routes of Aβ elimination being vascular-mediated clearance systems, including transcytosis and glymphatic/lymphatic drainage. Thus, there is increasing interest in treatments based on promoting vascular-mediated Aβ elimination.

### Transcytosis

Transcytosis in the cerebral capillaries is considered a major route of material exchange between the blood and the brain. The capillary lumen and brain parenchyma are separated by the BBB, which is composed of endothelial cells connected by tight junctions (51). The BBB prevents passive exchange between the blood and brain, and only allows controlled carrier-mediated bidirectional transport of nutrients and waste products (52).

Transcytosis is a major Aβ elimination system, and several molecules have been reported to be involved in Aβ transcytosis. Endothelial low-density lipoprotein receptor related protein-1 (LRP-1), a multifunctional scavenger and signaling receptor, is expressed in neural cells and cerebral microvessels (49, 53). Aβ binds to LRP-1 at the abluminal side of the endothelium, either as a free peptide or bound via ApoE2 and ApoE3. Aβ-ApoE2 and Aβ-ApoE3 complexes are rapidly cleared across the BBB into the blood, while Aβ bound to ApoE4 interacts poorly with LRP-1, and is removed from the brain by much slower and less efficient very low-density lipoprotein receptor clearance mechanism. Aβ transcytosis via LRP-2 was facilitated by clusterin in the brain (54–56). The importance of LRP-1 has also been shown by genetic linkage of LRP-1 with AD (57–59), and by the co-localization of LRP-1 with Aβ in senile plaques (60). LRP-1 staining in vessels was reduced in AD patients (49, 60). The transcytosis of Aβ was reported to be regulated by the phosphatidylinositol-binding clathrin assembly (PICALM) protein, a known genetic risk factor for AD (61).

The receptor for advanced glycation end products (RAGE), an immunoglobulin supergene family member, is another key molecule in Aβ transcytosis (62). Strong staining for RAGE was reported in the vessels of AD patients (60, 63), and RAGE was shown to mediate influx of circulating Aβ into the brain across the BBB (64). RAGE expression in microglia is also involved in microglial activation induced by Aβ (63, 65, 66). These findings prompted researchers to target RAGE for the treatment of AD. For example, inhibition of RAGE ameliorated cerebral Aβ burden and normalized cognitive performance in APP transgenic mice (67). A phase II clinical trial of the RAGE inhibitor TTP488 targeting mild-to-moderate AD patients was also associated with improved cognitive function as assessed by Alzheimer's Disease Assessment Scale-cognitive subscale (ADAS-cog) (68). However, a phase III trial was recently terminated as there was no positive effect using TTP488 treatment (69).

### Glymphatic and Lymphatic Drainage

The central nervous system was previously thought to be devoid of systemic lymphatic vessels. However, two recent investigations have clearly shown the presence of lymphatic vessels adjacent to meningeal vessels (70, 71). Ablation of meningeal lymphatic vessels also aggravated Aβ accumulation in AD model mice, suggesting that promoting intracranial lymphatic systems may be a potential therapeutic approach for AD (72).

**Figure 1:**
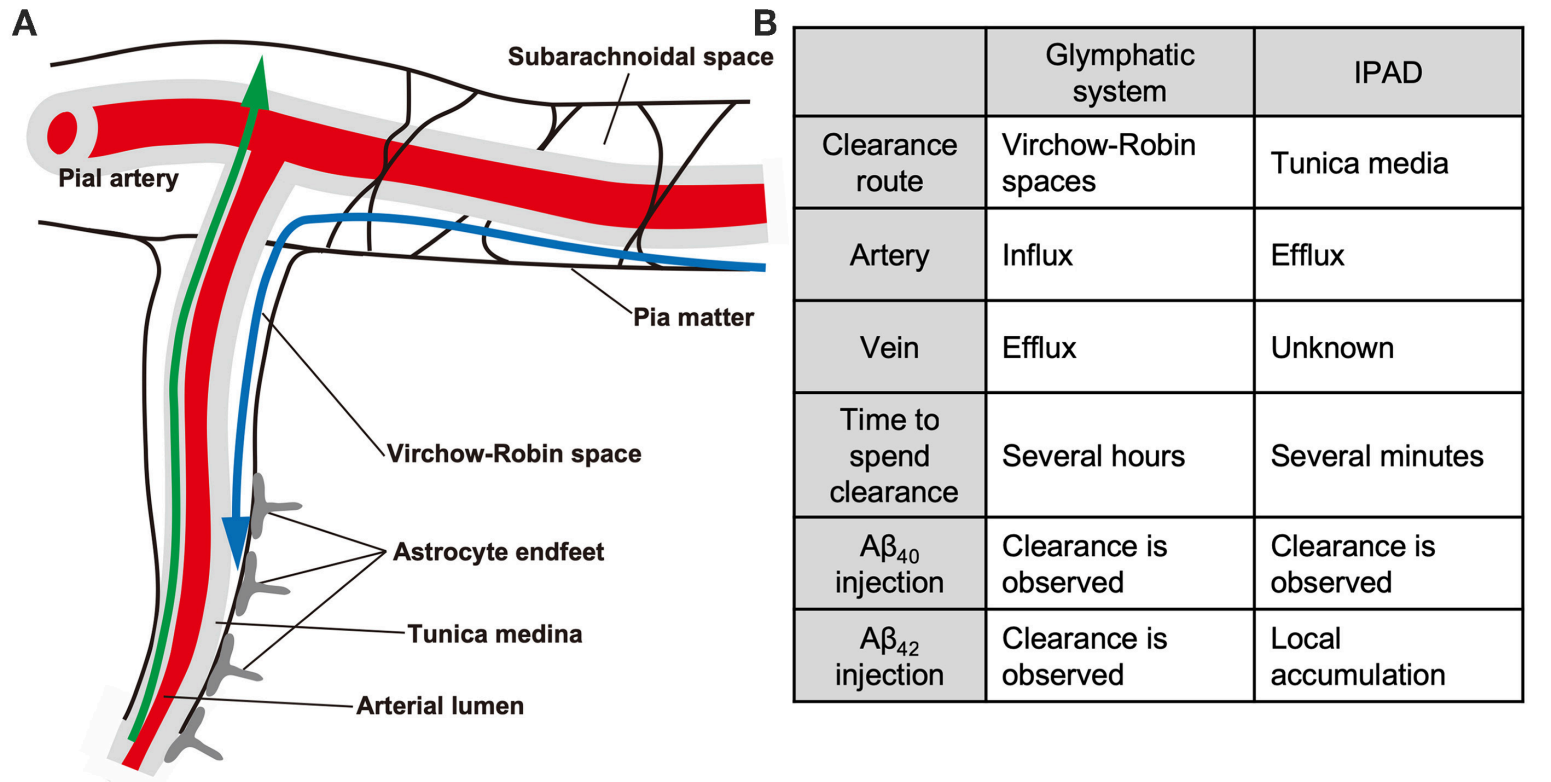
The glymphatic and IPAD systems. Schematic **(A)** and differences **(B)** of the glymphatic and IPAD systems. Note that the directions of the two clearance systems are the opposite. Blue, glymphatic system; green, IPAD. The IPAD is a more rapid clearance system than the glymphatic system. Note the differences in the tracer distribution pattern after fluorescent Aβ_40_ and Aβ_42_ injection.

The glymphatic paravascular drainage system is considered one of the Aβ clearance systems (Figure 1), consisting of the para-arterial CSF influx, convective bulk fluid flux, and para-venous interstitial fluid (ISF) efflux routes (73, 74). Tracers injected into the cisterna magna were found to gradually flow into the brain parenchyma through the perivascular space (PVS) around the arteries, and then flow out along the veins (75, 76). The PVS (also known as Virchow-Robin spaces) is a gap between the brain parenchyma derived from the ectoderm and the cerebral perforating arteries derived from the mesoderm. The fluid circulation from the subarachnoid space to the PVS was initially described in the 1980s (77). At first, the paravascular drainage was considered to be dependent on astroglial water transport via the astrocytic aquaporin-4 (AQP-4) water channel, because CSF influx was severely impaired in *Aqp-4* knockout mice (75). However, in a replica study, *Aqp-4* gene deletion did not affect fluid transport (78). In addition, the PVS is separated from the subarachnoid space by the pia matter (79, 80), and the degree to which these spaces communicate with each other remains unclear. Further studies are required to unravel the molecular mechanism of the glymphatic systems.

Clinical MRI often reveals an enlarged PVS in the basal ganglia in patients with hypertensive arteriopathy (81–84). Morphological changes in the PVS were also reported in patients with hereditary arteriopathy including Cerebral Autosomal Dominant Arteriopathy with Subcortical Infarcts and Leukoencephalopathy (CADASIL) (85) and Cerebral Autosomal Recessive Arteriopathy with Subcortical Infarcts and Leukoencephalopathy (CARASIL) (86). Both sporadic and hereditary arteriopathies are cerebral small vessel diseases (SVD), and expansion of the PVS is considered an imaging marker of SVD (87, 88). The presence of a large PVS was associated with a steeper decline in information processing speed, and an increased risk of developing vascular dementia (89). AD patients were also reported to show an increased ratio of total PVS to white matter volume (90). A mouse model of AD showed suppression of glymphatic paravascular fluid transport caused by accumulation of toxic Aβ (91), suggesting that PVS enlargement in AD patients may be associated with an impaired glymphatic system. An enlarged PVS is commonly observed in the centrum semiovale in both AD and cerebral amyloid angiopathy (CAA) (83, 84). CAA is another subtype of SVD observed in >80% of AD patients, as well as in ~10% of vascular cognitive impairment cases with a general absence of AD pathology (92, 93). After being generated in neurons, Aβ accumulates in the vessel walls (45), resulting in degeneration of smooth muscle cells, vessel wall thickening, luminal narrowing, and concentric wall splitting (double barreling) (94). The degeneration of mural cells and injury to vascular endothelial cells cause various forms of CVD, including ischemic stroke, hemorrhage, and white matter changes. Impaired BBB function likely interferes with Aβ transcytosis, leading to exacerbation of AD and CAA (21, 52).

The driving force behind paravascular clearance is thought to involve cerebral arterial pulsation. Indeed, reduced arterial pulsatility by unilateral carotid artery ligation prevented CSF-ISF exchange, while the opposite effect was observed following administration of the adrenergic agonist dobutamine (95). However, no studies have clearly shown that promotion of the glymphatic system can ameliorate Aβ pathology and cognitive function. In addition, several recent studies argued against the proposed glymphatic mechanism (78, 96–98). As Aβ rarely accumulates in the venous system, and as arterial Aβ accumulation is most prominent within the tunica medina, not the PVS (99), it remains unclear whether promotion of the glymphatic pathway may be a disease-modifying therapy for AD and CAA. Recent technological advances in imaging have enabled visualization and monitoring of PVS structure and change in AD patients (90, 100, 101). Clinical evaluation of the glymphatic system in AD patients may help to determine the relationship between the glymphatic system, PVS, and CAA.

Aβ distribution in CAA closely corresponds to the intramural periarterial drainage (IPAD) route, suggesting that congestion of this drainage pathway plays a major role in the pathogenesis of CAA (102). The IPAD system is also termed perivascular lymphatic drainage (103). ISF and solutes including Aβ are thought to be cleared from the gray matter through the IPAD, which is a space between two basement membranes in the walls of the cerebral capillaries and arteries (104, 105). Interestingly, IPAD was impaired in the aging mouse brain and in the presence of CAA (106). It was also reported that the IPAD and glymphatic systems are not independent, but rather communicate with each other (107). For example, Aβ tracer injected into the cisterna magna flowed into the PVS, while after reaching the deep brain parenchyma, some of the tracer drained out of the brain via the IPAD system. Because use of animal models with tracer injection is far from physiological, the precise relationship between the glymphatic system and IPAD remains elusive, and further studies are required.

The importance of the IPAD was demonstrated in a clinical trial of Aβ immunization. In AN-1792 vaccinated AD patients, parenchymal Aβ plaque was diminished while cerebrovascular Aβ accumulation was increased (108, 109). Considering that decreasing CSF Aβ was correlated with severity of CAA-causing CVD, the ineffectiveness and side-effects of AN-1792 may be partially explained by the excessive antibody-solubilized senile plaque Aβ that is re-deposited in the cerebral vasculature, and impaired Aβ clearance through IPAD (110, 111). Combination therapy of Aβ immunization and promoting Aβ clearance through IPAD may provide a solution for the problem in removal of solubilized Aβ from the brain. The fact that cerebral Aβ clearance was delayed after middle cerebral artery occlusion (112) and after bilateral common carotid artery stenosis (113) emphasizes the importance of cerebrovascular integrity for the promotion of Aβ clearance through IPAD.

### Promoting IPAD by Cilostazol

Cilostazol is a type 3 phosphodiesterase (PDE) inhibitor that is currently prescribed for secondary prevention of ischemic stroke and peripheral vascular disease, especially in Eastern Asia. PDE-3 can hydrolyze both cAMP and cGMP, while increasing cAMP level is a major pharmacological action of cilostazol (48). PDE-3 is widely expressed in the central nervous system, and is up-regulated in Aβ-positive vessels, especially in vascular smooth muscle cells (114), suggesting that PDE-3 inhibition may be an effective target for treatment of AD and CAA.

We previously demonstrated that cilostazol promoted IPAD, resulting in maintenance of vascular integrity, amelioration of Aβ deposits, and prevention of cognitive decline in AD model mice (114). Further, cilostazol possesses a wide range of pleiotropic effects, including neurogenesis (115, 116), differentiation of oligodendrocyte precursor cells (117), inhibition of lipid peroxidation (118, 119), enhancement of cholesterol efflux from macrophages (120), amelioration of insulin resistance (121), reduction of inflammatory burden (122–124), stabilization of BBB function (125), and improvement of systemic lymphatic function by inducing proliferation and stabilization of lymphatic endothelial cells (126). A nationwide cohort study in Taiwan showed that cilostazol treatment was associated with reduced risk of dementia (127). Favorable effects were also described in observational studies, which demonstrated the efficacy of cilostazol in patients with mild cognitive impairment (128) and AD (129–132). A phase-II, randomized clinical trial (the COMCID study) was started in 2015 (133), in which MCI patients received 100 mg daily cilostazol or placebo for 96 weeks. The results will be announced in 2020.

## Conclusions

AD, especially in the elderly, is a syndrome concomitant with CVD and neurodegeneration. The commonly used expression amongst clinicians, “a man is as old as his arteries,” is thus as relevant to patients with dementia as for many other age-related disorders (134). The influence of vascular factors should be given more consideration, as it may represent a future therapeutic target for dementia. The existence of multiple Aβ elimination mechanisms suggests the requirement for several different treatment approaches in AD. Further studies are essential for the development of novel treatments for dementia.

## Author Contributions

SS and YY wrote the manuscript. MI supervised and made critical revision of manuscript for important intellectual content.

### Conflict of Interest Statement

The authors declare that the research was conducted in the absence of any commercial or financial relationships that could be construed as a potential conflict of interest.
